# Clinical Predictors of Wheezing Among Children Infected With *Mycoplasma Pneumoniae*

**DOI:** 10.3389/fped.2021.693658

**Published:** 2021-09-22

**Authors:** Kaimeng Kong, Ying Ding, Beirong Wu, Min Lu, Haoxiang Gu

**Affiliations:** Department of Respiratory Medicine, Shanghai Children's Hospital, Shanghai Jiao Tong University, Shanghai, China

**Keywords:** wheezing, *Mycoplasma pneumonia* (MP), chest radiography, infiltration, allergy

## Abstract

**Background:***Mycoplasma pneumoniae* (MP) not only was a common pathogen of respiratory tract infections, but also could trigger the exacerbation of asthmatic symptoms in children with or without asthma.

**Objective:** This study aimed to identify possible risk factors associated with wheezing among children diagnosed with MP infection.

**Methods:** A retrospective analysis of medical records of children aged 28 days to 18 years old who visited the Shanghai Children's Hospital between January 2019 and January 2020 was carried out, and all children were then classified into three groups: two wheezing groups (with or without MP infection) and a non-wheezing group with MP infection. Information including patient's demographics, clinical features, laboratory data, and radiography findings was extracted from the electronic medical record system. Chest radiographs were reviewed independently by two board-certified, blinded pediatric radiologists.

**Results:** A total of 1,512 patients were included in our study, and 21.9% of them belonged to the wheezing group without MP infection. Among 1,181 patients with MP infection, 295 people (25.0%) suffered from wheezing, and males accounted for 61%. Through the multivariable logistic regression analyses, we found that six variables were positively associated with wheezing attacks in children with MP infection: male gender (likelihood ratio [LR] = 2.124, 95% confidence interval [CI]: 1.478–3.053), history of allergy (LR= 3.301, 95% CI: 2.206–4.941), history of wheezing (LR = 7.808, 95% CI: 5.276–11.557), autumn in reference to summer (LR = 2.414, 95% CI: 1.500–3.885), non-end-point infiltration in reference to consolidation or pleural effusion (LR = 1.982, 95% CI: 1.348–2.914), and infiltration scope (LR = 1.773, 95% CI: 1.293–2.432). However, the model showed that the probability of wheezing after MP infection decreased as age increased (LR = 0.257, 95% CI: 0.196–0.337). Moreover, the area under the curve (AUC) of the regression model was as high as 0.901 (0.847–0.955).

**Conclusion:** The model integrated with factors including gender, age, season, radiological patterns, infiltration scope, and history of allergy performed well in predicting wheezing attack after MP infection in children.

## Introduction

As a type of mucosal pathogen, *Mycoplasma pneumoniae* (MP) colonizes and infects epithelial cells of the respiratory tract, and can penetrate the plasma membrane of host cells to invade the respiratory mucosa, thus leading to respiratory disease (1). The pathogenesis of MP infection involves direct damage and immune-mediated damage mechanisms, including adhesion damage, membrane fusion damage, immune damage, inflammation damage, and so on (1, 2). MP is a common respiratory pathogen in children and can cause rhinitis, otitis, pharyngitis, and bronchitis, as well as community-acquired pneumonia (3, 4).

In recent years, more and more evidence have showed that MP infection involved a wide range of disease in addition to respiratory diseases, such as hemolytic anemia, myocarditis pericarditis, and acute hepatitis, which are also termed MP-related extra-pulmonary diseases (MpEPDs) (5, 6).

It has been reported that a third of infants experienced multiple episodes of wheezing during their first 3 years, and nearly half of the children have wheezed before the age of 6 years (7). Childhood wheezing is the most common manifestation of asthma onset in relation to the lung function of older children (8).With the increasing incidence of MP infection, an equal number of studies have found an association between MP infection and wheezing (9–12). MP infection may not only trigger the exacerbation of asthmatic symptoms but also can be accompanied by wheezing in children who were never diagnosed with asthma (9, 10, 12). Previous research provides an interpretation, as MP infection has relevance to persistent bronchial hyper-responsiveness and chronic airway inflammation (13). However, not all children with MP infection have exacerbation of wheezing. Additionally, associations between host factors, including developing immune and respiratory systems, as well as individual genetic variations and wheezing in children with MP infection, have yet to be clearly elucidated. Therefore, we examined whether clinical characteristics, laboratory findings, and radiography findings were associated with wheezing in children with MP infection in a retrospective study.

## Methods

### Study Subjects

This descriptive, retrospective study reviewed clinical records of children diagnosed with MP infection at the Shanghai Children's Hospital between January 2019 and January 2020. Meanwhile, wheezing children without MP infection during the same period were selected as a control group.

Inclusion criteria were patients aged ≥28 days and ≤18 years old with MP infection or wheezing children without MP infection, who may had an infection mixed with other pathogens. Children were excluded if they met any of the following conditions: (1) congenital malformations of the lungs and airways, (2) presence of a tracheal foreign body, (3) bronchopulmonary dysplasia, (4) congenital heart disease with hemodynamic changes, and (5) incomplete medical records. Patients were classified into three groups: a wheezing group without MP infection, a wheezing group with MP infection, and a non-wheezing group with MP infection. Wheezing was defined according to whether doctors heard a high-pitched, whistling sound from the chest during the expiratory phase throughout the course of the disease, and the information was retrieved through the clinic medical record and hospital medical record by a researcher who was blinded to the whole experimental design. The current study was conducted with approval from the Ethics Committee of the Children's Hospital of Shanghai (2017R016-F01). Verbal consent was obtained from the guardian of all children through follow-up phone calls.

### Definition of Acute MP Infection

Throat swabs taken from all children at admission were assessed by real-time polymerase chain reaction (RT-PCR) for the presence of MP. Samples for RT-PCR were obtained from oropharynx at the posterior pharyngeal wall with a cotton-tipped swab and transferred to a tube containing a commercial transport medium (Copan Italia SPA, Brescia, Italy). The tube was then transported to the laboratory on ice and stored at −70°C until PCR processing. RT-PCR was performed using an Applied Biosystems 7500 Real-Time PCR system (Applied Biosystems, Foster City, USA). Blood samples were obtained from all children at admission, during hospitalization, or 2 weeks thereafter in the outpatient. MP-specific IgM and IgG antibodies were determined using a commercially available passive particle agglutination kit (Diagnostic Kit for Measurement of Antibodies to *M. pneumoniae*, SERODIA ® -MYCO II).

Acute MP infection was diagnosed if at least one test was positive (detection of MP by PCR or detection of MP-specific antibodies by serology). Serological diagnosis was defined when specific IgG antibodies were detected (≥1:160) in a single serum sample or if an increase of at least four fold in the specific IgG antibody titer in the acute phase or recovery phase. In addition, acute MP infection was also diagnosed if the PCR performed on nasopharyngeal aspirates was positive for DNA (14).

### Extraction of Medical History Information

Information including clinical characteristics, laboratory data, and radiography findings was retrieved from an electronic medical history system. Demographic sociology characteristics included gender; age; weight; birth weight; household cigarette smoking; family history of allergy; history of allergy including asthma, allergic rhinitis, and/or atopic dermatitis; breastfeeding; history of wheezing; allergen sensitization; and admission season. Laboratory data consisted of the mycoplasma-specific antibody titer; the presence or absence of mycoplasma DNA; a routine blood test covering leucocytes (×10^9^/L), neutrophils (%), lymphocytes (%), eosinophils (%), hemoglobin levels (g/L), platelets (×10^9^/L), POCT (mg/L), serum total IgG (g/L), serum total IgA (g/L), serum total IgM (g/L), serum total IgE (g/L), and the ratio of CD4^+^/CD8^+^ cells; whether complications from viral infection were present; and whether macrolide-resistant mycoplasma or severe pneumonia was present. Imaging data included chest x-rays or computerized tomography findings.

According to age distribution, we divided all subjects into the following three groups: infants (>28 days and <3 years old), pre-school-age children (≥3 and <6 years old), and school-age children (≥6 and ≤18 years old). Classification of body weight and severe pneumonia was defined according to weight-for-age *z*-scores (15) and World Health Organization (WHO) criteria (16), respectively.

### Radiography Classifications

Radiography findings consisted of chest x-rays or computerized tomography findings taken during outpatient service or hospitalization. Chest radiographs were independently analyzed by two board-certified, blinded pediatric radiologists, and were divided into three categories based on WHO criteria as follows: class I, primary end-point consolidation or pleural effusion; class II, non-end-point infiltrate; and class III, no consolidation, infiltrate, or effusion (17). According to the scope of infiltration, all imaging data were classified into three classes: no infiltration, unilateral infiltration, and bilateral infiltration.

### Statistical Analysis

Descriptive statistics were performed on clinical data of the study population and are expressed as mean ± standard deviation (SD) for continuous variables and percentage for categorical variables. Independent-sample *t*-tests were performed if continuous variables followed a normal distribution; otherwise, nonparametric Mann–Whitney *U* tests were performed. χ^2^ tests were conducted for categorical data. Considering that some laboratory data were age-independent variables, we performed a stratification analysis according to age classification. Variables with *p* < 0.05 in univariate analysis were further included into the stepwise logistic regression model, and odds ratio (OR) and its 95% CIs was calculated. Moreover, receiver operating characteristics (ROC) analysis was used to assess the accuracy of the logistic regression model and single variable in predicting wheezing after MP infection. Statistical analyses were conducted using SPSS software 20.0, and all tests were two-sided, with *p* < 0.05 being considered statistically significant.

## Result

### Clinical Characteristics of Population

A total of 1,512 patients were ultimately included in this study, and 331 patients belonged to the wheezing group without MP infection. Among 1,181 patients with MP infection, 295 people (25.0%) suffered from wheezing, and the male gender accounted for 61%, which was significantly higher than that of the non-wheezing group but lower than that of the wheezing group without MP infection. The average age of patients in the wheezing group with MP infection was 3.7 years (SD = 2.3 years), which was younger than that of the non-wheezing group (6.12 ± 0.7 year), but was older than that of the wheezing group without MP infection.

Percentages of male children and infants in the wheezing group without MP infection were 71.3 and 70.7%, respectively, which were significantly higher than those of the other two groups. The incidence of wheezing in children with a family history of allergy or a personal history of allergy was significantly increased after MP infection, and the difference between the two groups was statistically significant (both *p* < 0.001). In addition, 161 of the 295 (54.6%) wheezing patients with MP infection had a history of wheezing, as against only 87 of the 886 (9.8%) non-wheezing patients with MP infection and 149 of the 331 (45.0%) wheezing patients without MP infection (both *p* < 0.001). Compared with the other three seasons, the peak incidence of wheezing was in autumn, and the difference was statistically significant between the wheezing and non-wheezing group with MP infection (*p* < 0.001), but the occurrence of wheezing without MP infection was mainly in winter. No significant differences existed for factors including birth weight, inhaled or food allergen sensitization, household cigarette smoking, or breastfeeding between wheezing and non-wheezing groups with MP infection. Clinical characteristics of all included patients with and without wheezing are summarized in Table 1.

**Table 1:** Clinical characteristic of all included patients with and without MP infection.

**Clinical characteristics**	**Wheezing group without MP infection (*n* = 331)**	**MP infection groups**		
		**Wheezing (*n* = 295)**	**Non-wheezing (*n* = 886)**	***p*-value**
Male, *n* (%)	236 (71.3%)^**※**^	180 (61.0%)	397 (44.8%)	<0.001
Age (years)	2.7 ± 1.9^**※**^	3.7 ± 2.3	6.1 ± 2.7	<0.001
Age groups				
Infants, *n* (%)	234 (70.7%)^**※**^	138 (46.8%)	109 (12.3%)	
Pre-school age, *n* (%)	82 (24.8%)	112 (38.0%)	316 (35.7%)	<0.001
School age, *n* (%)	15 (4.5%)	45 (15.2%)	461 (52.0%)	
Weight classifications				
Under weight, *n* (%)	28 (8.5%)	41 (13.9%)	87 (9.8%)	
Normal weight, *n* (%)	295 (89.1%)	251 (85.1%)	773 (87.2%)	0.033
Overweight/Obese, *n* (%)	8 (2.4%)	3 (1.0%)	26 (3.0%)	
Birth weight				
Low birth weight, *n* (%)	18 (5.4%)	17 (5.8%)	28 (3.2%)	
Normal birth weight, *n* (%)	285 (86.1%)	256 (86.8%)	808 (91.2%)	0.061
High birth weight, *n* (%)	28 (8.5%)	22 (7.4%)	50 (5.6%)	
Inhaled allergen sensitization, *n* (%)	43 (13.0%)	41 (13.9%)	141 (15.9%)	0.491
Food allergen sensitization, *n* (%)	70 (21.1%)	52 (17.6%)	128 (14.4%)	0.816
Household cigarette smoking, *n* (%)	63 (19.0%)^**※**^	77 (26.1%)	184 (20.8%)	0.056
Family history of allergy, *n* (%)	112 (33.8%)	106 (35.9%)	205 (23.1%)	<0.001
History of allergy, *n* (%)	97 (29.3%)^**※**^	123 (41.7%)	169 (19.1%)	<0.001
Breastfeeding, *n* (%)	181 (54.7%)^**※**^	160 (54.2%)	464 (52.4%)	0.578
History of wheezing, *n* (%)	149 (45.0%)^**※**^	161 (54.6%)	87 (9.8%)	<0.001
Admission season				
Spring, *n* (%)	43 (13.0%)^**※**^	63 (21.4%)	238 (26.9%)	
Summer, *n* (%)	92 (27.8%)	37 (12.5%)	186 (21.0%)	
Autumn, *n* (%)	73 (22.1%)	125 (42.4%)	277 (31.3%)	<0.001
Winter, *n* (%)	123 (37.2%)	70 (23.7%)	185 (20.8%)	

※ *Statistical difference existed between wheezing groups with and without MP infection*.

### Laboratory Findings

Among all children with MP infection, no differences existed in white blood cell count, neutrophils/lymphocytes/eosinophil/basophil ratios, and blood platelet counts between wheezing and non-wheezing groups as determined in the age-stratification analysis. The hemoglobin level for wheezing children was 123.41 ± 9.46 g/L (mean ± standard deviation), which was significantly higher than that for the non-wheezing children (121.23 ± 9.33 g/L, *p* = 0.035) in the pre-school-age stratification, but no difference existed in the other two age stratifications. The serum POCT level was 10.65 ± 14.54 (mg/L) in the wheezing group, which was significantly below that of the non-wheezing group (14.10 ± 17.02 mg/L, *p* = 0.022) of infants.

For immunoglobulin levels, serum total IgG, IgA, and IgM levels did not show significant *p*-values and could not be used to distinguish wheezing from not wheezing in the three groups stratified for age. However, the serum total IgE level in the wheezing group was significantly higher than that of the non-wheezing group in pre-school-age and school-age children. School-age children having a higher ratio of CD4^+^/CD8^+^ cells were inclined to wheeze (*p* = 0.001), but no differences existed between wheezing and non-wheezing groups in infants and pre-school-age children.

No difference was found between wheezing and non-wheezing groups for the incidence of severe pneumonia, virus infection, or drug-resistant MP, as well as MpEPDs in pre-school-age and school-age groups. MP infection combined with MpEPD was negatively correlated with the occurrence of wheezing in the infant group; however, this correlation was not found in pre-school-age or school-age groups. Laboratory findings of MP-infected patients with and without wheezing are shown in Table 2.

**Table 2:** Laboratory findings of MP-infected patients with and without wheezing.

**Variables**	**Infants**		**Pre-school age children**		**School age children**	
	**Wheezing** **(*n* = 138)**	**Non-wheezing** **(*n* = 109)**	**Wheezing** **(*n* = 112)**	**Non-wheezing** **(*n* = 316)**	**Wheezing** **(*n* = 45)**	**Non-wheezing** **(*n* = 461)**
White blood cell (×10^9^/L)	9.47 ± 3.93	9.31 ± 4.45	7.90 ± 2.92	7.69 ± 3.59	7.83 ± 3.31	7.30 ± 3.07
Neutrophils (%)	48.26 ± 18.72	44.44 ± 18.28	56.32 ± 16.42	55.70 ± 14.00	64.92 ± 12.88	62.90 ± 12.24
Lymphocytes (%)	43.15 ± 17.12	44.99 ± 17.23	34.26 ± 14.53	34.59 ± 12.93	24.77 ± 10.80	27.25 ± 10.93
Eosinophil (%)	1.37 ± 1.85	2.04 ± 2.36	1.93 ± 2.00	1.80 ± 2.33	2.66 ± 2.76	2.25 ± 2.53
Basophil (%)	0.18 ± 0.12	0.19 ± 0.15	0.22 ± 0.15	0.22 ± 0.16	0.23 ± 0.17	0.24 ± 0.16
Hemoglobin (g/L)	121.91 ± 9.18	120.74 ± 9.91	123.41 ± 9.46	121.23 ± 9.33^**※**^	125.87 ± 9.38	124.49 ± 9.17
Blood platelet (×10^9^/L)	376.65 ± 136.97	344.12 ± 127.35	341.09 ± 115.93	320.76 ± 103.49	324.18 ± 97.44	313.58 ± 105.36
POCT (mg/L)	10.65 ± 14.54	14.10 ± 17.02 ^**※**^	14.15 ± 12.68	17.75 ± 21.34	19.20 ± 23.09	20.24 ± 22.09
IgE (g/L)	293.19 ± 846.85	266.11 ± 507.47	340.85 ± 596.24	228.30 ± 304.47^**※**^	627.27 ± 940.13	327.45 ± 495.32^**※**^
IgG (g/L)	7.97 ± 2.05	8.24 ± 2.23	9.11 ± 2.28	9.43 ± 2.06	11.10 ± 2.46	10.87 ± 2.32
IgM (g/L)	1.45 ± 0.55	1.58 ± 0.60	1.65 ± 0.55	1.69 ± 0.83	1.93 ± 1.06	1.82 ± 0.93
IgA (g/L)	0.70 ± 0.43	0.74 ± 0.42	1.07 ± 0.51	1.13 ± 0.50	1.75 ± 0.84	1.63 ± 0.67
CD4/CD8	1.70 ± 0.67	1.72 ± 0.66	1.39 ± 0.42	1.47 ± 0.50	1.51 ± 0.46	1.34 ± 0.45^**※**^
Severe pneumonia, *n* (%)	33 (23.91%)	32 (29.36%)	35 (31.25%)	114 (36.08%)	12 (26.67%)	182 (39.48%)
With virus infection, *n* (%)	68 (49.28%)	47 (43.12%)	57 (50.89%)	131 (41.46%)	17 (37.78%)	130 (28.20%)
Drug resistant MP, *n* (%)	58 (42.03%)	33 (30.28%)	52 (46.43%)	155 (49.05%)	25 (55.56%)	239 (51.84%)
With MpEPDs, *n* (%)	22 (15.94%)	35 (32.11%) ^**※**^	20 (17.86%)	54 (17.09%)	4 (8.89%)	96 (20.82)

※ *Statistical difference existed between wheezing and non-wheezing groups infected with MP*.

Two hundred and thirty-one children were diagnosed with MpEPDs, and no difference was found for the incidence of MpEPDs, serum total IgE level, ratio of CD4^+^/CD8^+^ cells, and eosinophil (%) among the three groups. Infants with a history of allergy had a higher incidence of MpEPDs than that of the group without a history of allergy (24.6 vs. 12.1%, *p* = 0.033), and the percentage of wheezing for infants with MpEPDs was 38.6%, which was significantly lower than that of infants without MpEPDs (61.1%, *p* = 0.004). Comparison of clinical characteristics and laboratory findings in patients with and without MpEPDs is shown in Table 3.

**Table 3:** Comparison of clinical characteristic and laboratory findings in patients with and without MpEPDs.

**Variables**	**Infants**		**Pre-school-age children**		**School-age children**	
	**With MpEPDs** **(*n* = 57)**	**Without MpEPDs (*n* = 190)**	**With MpEPDs** **(*n* = 74)**	**Without MpEPDs (*n* = 354)**	**With MpEPDs** **(*n* = 100)**	**Without MpEPDs (*n* = 406)**
Eosinophil (%)	2.152 ± 0.48	1.521 + 0.97	1.301 ± 0.34	1.942 ± 0.38	2.272 ± 0.94	2.292 ± 0.45
IgE (g/L)	203.42 ± 328.56	304.59 ± 795.53	276.78 ± 357.81	253.77 ± 413.41	288.43 ± 381.42	370.29 ± 588.97
CD4/CD8	1.70 ± 0.64	1.70 ± 0.67	1.48 ± 0.53	1.44 ± 0.47	1.32 ± 0.50	1.37 ± 0.44
History of allergy, *n* (%)	14 (24.6%)	23 (12.1%)^**※**^	20 (27.0%)	102 (28.8%)	22 (22.0%)	111 (27.3%)
Inhaled allergen sensitization, *n* (%)	11 (19.3%)	24 (12.6%)	10 (13.5%)	38 (10.7%)	15 (15.0%)	71 (17.5%)
Food allergen sensitization, *n* (%)	7 (12.3%)	26 (13.7%)	14 (18.9%)	64 (18.1%)	15 (15.0%)	67 (16.5%)
Wheezing episode, *n* (%)	22 (38.6%)	116 (61.1%)^**※**^	20 (27.0%)	92 (26.0%)	4 (4.0%)	41 (10.1%)

※ *Statistical difference existed between groups with and without MpEPDs*.

### Chest Radiographs

Among all children infected with MP, abnormalities in chest radiographs were found in 1,165 children (98.6%), including consolidation, infiltration, and effusion, featuring peribronchial thickening and multiple areas of atelectasis according to WHO classification (17). There were 524 cases, 442 cases, and nine cases receiving CT scan in WHO Class I group, Class II group, and Class III group, respectively. Two hundred and twenty-one wheezing children with MP infection were classified into WHO Class II group, which consisted of minor patchy infiltrates (205 cases), featuring peribronchial thickening (13 cases), pulmonary atelectasis (one case), and local pulmonary fibrosis (two cases). Seventy-one wheezing children with MP infection belonged to WHO Class I group, and chest imaging of 56 children were just end-point consolidation. Percentage of wheezing children showing non-end-point infiltrate in chest imaging was 74.9%, which was significantly higher than that of the non-wheezing group (47.0%), and there was statistical significance among the three WHO radiographic classifications groups in the incidence of wheezing (*p* < 0.001). Of 1,126 cases identifying with lung infiltration, 46.0% had a single infiltration. Two hundred and nine children with bilateral infiltration had wheeze onset, which was higher than the numbers in the other two groups (*p* < 0.001). Table 4 showed chest radiographs of MP-infected children with and without wheezing.

**Table 4:** Chest radiographs of MP-infected children with and without wheezing.

**Variables**	**Groups**		***p*-value**
	**Wheezing (*n* = 295)**	**Non-wheezing (*n* = 886)**	
WHO Radiological patterns			
Class I	71 (24.1%)	457 (51.6%)	
Class II	221 (74.9%)	416 (47.0%)	<0.001
Class III	3 (1.0%)	13 (1.4%)	
Infiltration scope			
Neither	17 (5.7%)	38 (4.3%)	
Unilateral	69 (23.4%)	449 (50.7%)	<0.001
Bilateral	209 (70.9%)	399 (45.0%)	

### Multiple Regression Analysis

**Figure 1:**
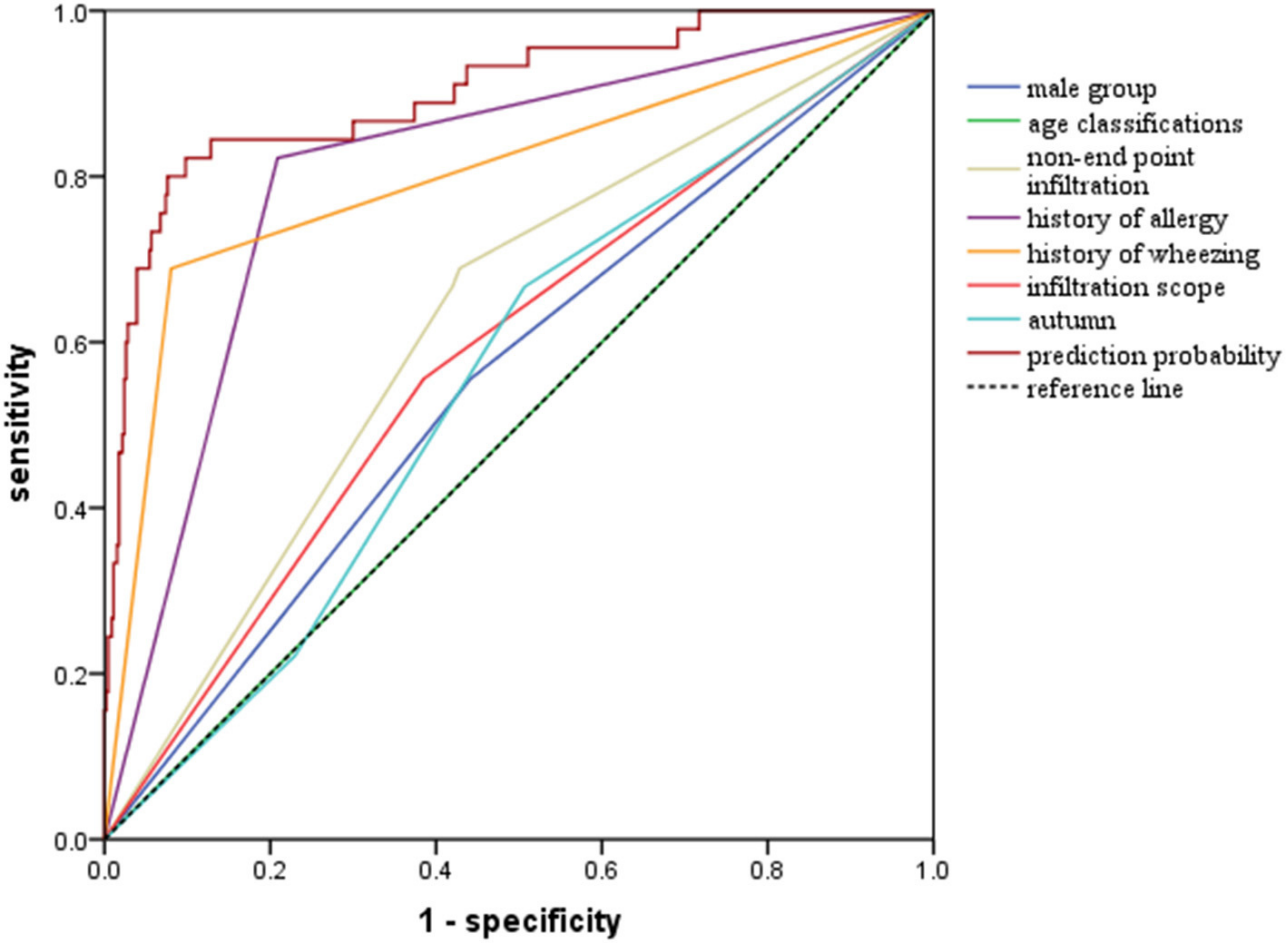
Receiver operator curves (ROC) of multiple logistic regression model and single univariate variable as predictors of wheezing among children with MP infection.

Univariate analysis showed that factors such as gender, age, family history of allergy, history of allergy, IgE levels, and so on could help to differentiate between wheezing and non-wheezing children with MP infection; therefore, we included the mentioned univariate into multiple logistic regression equation. However, multiple logistic regression indicated that only the male group (positive likelihood ratio [LR] = 2.124, 95% CI: 1.478–3.053), age (negative LR = 0.257, 95% CI: 0.196–0.337), history of allergy (positive LR = 3.301, 95% CI: 2.206–4.941), history of wheezing (positive LR = 7.808, 95% CI: 5.276–11.557), autumn (positive LR = 2.414, 95% CI: 1.500–3.885), non-end-point infiltration (positive LR = 1.982, 95% CI: 1.348–2.914), and infiltration scope (positive LR = 1.773, 95% CI: 1.293–2.432) were independent factors allowing differentiation between wheezing and non-wheezing individuals with MP infection. Table 5 shows independent odds ratios and confidence intervals for the effects of each variable on the risk of wheezing among MP-infected patients. The ROC analysis showed that the area under the curve (AUC) of the multiple logistic regression model was 0.901 (0.847–0.955), exceeding that of the single variable. ROC and AUC of the regression model and the single univariate variable as predictors of wheezing in children with MP infection are shown in Figure 1 and Table 6, respectively.

**Table 5:** Independent odds ratios and confidence intervals for the effects of each variable on the risk of wheezing among MP-infected patients.

**Variables**	**LR**	**95% Cis**	***p*-value**
Gender (reference: female)	2.124	1.478–3.053	<0.001
Age classifications	0.257	0.196–0.337	<0.001
History of allergy	3.301	2.206–4.941	<0.001
History of wheezing	7.808	5.276–11.557	<0.001
Season (reference: summer)			
Spring	0.886	0.492–1.598	0.689
Autumn	2.414	1.500–3.885	0.001
Winter	1.660	0.983–2.803	0.058
WHO radiological patterns (reference: primary end-point consolidation or pleural effusion)			
Non-end-point infiltration	1.982	1.348–2.914	<0.001
No consolidation/infiltrate/effusion	1.796	0.321–10.041	0.505
Infiltration scope	1.773	1.293–2.432	<0.001

**Table 6:** Area under the curve (AUC) of the multiple logistic regression model and the single univariate variable as predictors of wheezing in children with MP infection.

**Variables**	**AUC (95% CI)**	***p*-value**
Regression model	0.901 (0.847–0.955)	<0.001
History of wheezing	0.804 (0.722–0.886)	<0.001
Non-end point infiltration	0.629 (0.546–0.711)	0.004
Infiltration scope	0.586 (0.498–0.674)	0.057
History of allergy	0.807 (0.739–0.875)	<0.001
Male group	0.557 (0.469–0.645)	0.206
Autumn	0.557 (0.475–0.640)	0.204
Age classification	0.500 (0.412–0.588)	1.000

## Discussion

MP was closely associated with respiratory infections, particularly among school-aged children and young adults, but the prevalence of MP infection among younger children has been increasing in recent years (1, 3, 18). In our study, we found MP-associated wheezing accounted for 47.2% and mainly happened among young children; however, wheezing children with MP infection were still older than wheezing children without MP infection. Published literature shows that preschool children have a higher morbidity of wheezing, but the incidence of wheezing remits by the age of 6 years (19). Consistent with previous evidence, our study found that the incidence of wheezing after MP infection significantly decreased with age increasing (negative LR: 0.257 [95% CI: 0.196–0.337]). We found that male children were more inclined to experience wheezing than female children after MP infection. This may be because male children have a smaller airway with respect to lung volume, and they were more sensitive to airway irritants, such as respiratory infections or allergens (20). In our study, the positive LRs of history of allergy and wheezing were 3.301 (95% CI: 2.206–4.941) and 7.808 (95% CI: 5.276–11.557), respectively. We speculated that bronchial responsiveness of such population was high, and recurrent wheezing may harm the peripheral airway, which may explain why these populations were susceptible to wheezing when infected with MP. However, the medical information about the pathogen of previous wheezing was incomplete, so we could not infer the influence of previous pathogen on the occurrence of wheezing after MP infection.

The relationship between MP infection and asthma-like symptoms has attracted increasing attention in recent years. Individuals who have experienced wheezing usually exhibit airway hyper-responsiveness and inflammation, which possibly reduces the ability of the airway to eliminate MP infection (21). Several studies in the literature provide evidence that the interaction effect between the host immune response and cell- or cytokine-mediated in?ammation plays a significant role in the pathogenetic mechanism of MP infection, which is closely associated with the occurrence of wheezing (22–24). On the other hand, imbalance of the T-helper type 1 (T_H_1)–T-helper type 2 (T_H_2) immune response could trigger wheezing. T lymphocytes, especially those involved in the T_H_2 response, promote airway remodeling, chronic airway inflammation, and airway hyper-reactivity (25). Interleukin (IL)-4, IL-5, and IL-13, mainly secreted by T_H_2 cells, are the critical stimulus that promotes serum IgE production and are crucial players in the development of allergic disease and wheezing (26). All of these abovementioned conditions predispose the airway to MP infection and thus establish a vicious cycle. We found no difference in aspects including eosinophil, the CD4^+^/CD8^+^ ratio, serum total IgE, family history of allergy, and allergen sensitization between wheezing and non-wheezing groups in the multiple regression analysis (23). Previous evidence supportive of our findings is that wheezing-associated symptoms are correlated with serum IgE levels and with the existence of allergies in older children, but this relationship is not evident in younger children (27). Childhood wheezing is generally categorized into three phenotypes: early transient wheezing, late-onset wheezing, and persistent wheezing. Transient early wheezing occurs before the first 3 years of life, remits at the age of 6 years, and is related to respiratory tract infections. On the other hand, children with transient wheezing had no family history of asthma or allergen sensitization (28). In our study, children in wheezing groups no more than 6 years of age accounted for 84.8% of individuals; thus, we inferred that most wheezing episodes were related to early transient wheezing, which could account for our finding. Moreover, in our age-stratification analysis, we found that wheezing children in both pre-school-age and school-age groups had a higher serum IgE level than that of non-wheezing children, as well as a higher CD4^+^/CD8^+^ ratio in school-age children, which indicates that IgE plays a crucial role in the occurrence of wheezing in children no younger than 3 years old.

Previous literature showed that children diagnosed with MpEPDs had a significantly increased serum IgE level, and the occurrence of MpEPDs was correlated with atopy and/or respiratory allergy in children (6, 29, 30).

In our study, no association was found between the occurrence of MpEPDs and serum IgE level, eosinophil (%), the ratio of CD4^+^/CD8^+^ cells, as well as the existence of inhaled/food allergen sensitization. However, we found that infants with a history of allergy tend to be diagnosed with MpEPDs, but infants diagnosed with MpEPDs had a lower occurrence of wheezing, which further indicated that allergy-mediated mechanisms may not be largely involved in the occurrence of wheezing in younger children <3 years old.

Wheezing was a high-pitched, whistling sound that occurred during the extended expiratory phase because of the rapid passage of air through the narrowed airway. As a result, diseases affecting the peripheral airway are more likely to lead to wheezing. The association between wheezing and pneumonia has been widely examined and provides support that wheezing episodes predispose young children to pneumonia (31). However, limited research has explored the relationship between different radiological findings in pneumonia and wheezing. The prevalence of pneumonia in previous investigations conducted with wheezing children varied widely, from 1 to 23% (32, 33). However, in our study, the incidence of pneumonia was increased, which demonstrates that MP infection enhances the presence of pneumonia in wheezing individuals. In addition, we found that radiological findings of non-end-point infiltrate were associated with the highest incidence of wheezing among the three WHO classifications. In reference to the primary end-point consolidation/pleural effusion, radiological manifestations of non-end-point infiltrate (positive LR: 1.982 [95% CI: 1.348–2.914]) were associated with an increasing risk of wheezing. One interpretation is that consolidation of lung tissue significantly reduced or even completely blocked the entry of gas, thereby reducing the influence of highly resistant peripheral airways on the exhaled gas. Moreover, the probability of wheezing significantly increased (positive LR: 1.773 [95% CI: 1.293–2.432]), as the scope of infiltration increased. Along with our study, Hanhan et al. (34) revealed that the incidence of positive chest x-ray (infiltrates) was relatively higher in those asthmatics who had mycoplasma-specific IgM. Therefore, chest radiography to evaluate the presence of pneumonia was required for wheezing patients with MP infection, especially when the therapeutic effect was not satisfactory.

While previous studies have struggled to identify factors predicting wheezing after respiratory tract infection (35–37), one single factor could not accurately forecast the outcome, owing to potential confounders. Therefore, it seemed necessary to adopt multivariable analysis to seek independent indicators of wheezing. Indeed, our study emphasized that the discriminating capability of the regression model was superior to that of single characteristics in predicting exacerbations of wheezing after MP infection. The AUC of the model was as high as 0.901, which was higher than that of univariate variable. Moreover, among the separate variables, history of wheezing (AUC: 0.804 [95% CI: 0.722–0.886]) and history of allergy (AUC: 0.807 [95% CI: 0.739–0.875]) were considered as crucial determinants in predicting the occurrence of wheezing after MP infection.

## Conclusion

The present study highlights the combined effect of factors including gender, age, history of allergy, history of wheezing, season, radiological patterns, and infiltration scope on the occurrence of wheezing under conditions of MP infection in children. However, several limitations in our study should be considered. First, as a retrospective study, there may have been several selection bias. Secondly, the absence of information about previous pathogen in children with recurrent wheezing prevents us from exploring the impact of previous infection on the occurrence of wheezing after MP infection. Thirdly, failure to exclude the combination with viral or bacterial infections may affect the accuracy of our regression equation.

## Data Availability Statement

The raw data supporting the conclusions of this article will be made available by the authors, without undue reservation.

## Ethics Statement

The studies involving human participants were reviewed and approved by Ethics Committee of the Children's Hospital of Shanghai. Written informed consent to participate in this study was provided by the participants' legal guardian/next of kin. Written informed consent was obtained from the minor(s)' legal guardian/next of kin for the publication of any potentially identifiable images or data included in this article.

## Author Contributions

HG and KK were responsible for the conception and design of the study. KK and YD collected the data, completed the statistical analysis, and drafted the manuscript. BW and ML participated in valuable discussions. All authors reviewed and approved this manuscript.

## Conflict of Interest

The authors declare that the research was conducted in the absence of any commercial or financial relationships that could be construed as a potential conflict of interest.

## Publisher's Note

All claims expressed in this article are solely those of the authors and do not necessarily represent those of their affiliated organizations, or those of the publisher, the editors and the reviewers. Any product that may be evaluated in this article, or claim that may be made by its manufacturer, is not guaranteed or endorsed by the publisher.
